# Role of NT-proBNP and lung ultrasound in diagnosing and classifying heart failure in a hospitalized oldest-old population: a cross-sectional study

**DOI:** 10.1186/s12877-024-04977-4

**Published:** 2024-04-20

**Authors:** Matteo Landolfo, Francesco Spannella, Federico Giulietti, Chiara Di Pentima, Piero Giordano, Elisabetta Borioni, Laura Landi, Mirko Di Rosa, Roberta Galeazzi, Riccardo Sarzani

**Affiliations:** 1 Internal Medicine and Geriatrics, IRCCS INRCA, Via della Montagnola 81, 60127 Ancona, Italy; 2 Department of Clinical and Molecular Sciences, University Politecnica Delle Marche, Ancona, Italy; 3 Unit of Geriatric Pharmacoepidemiology and Biostatistics, IRCCS INRCA, Ancona, Italy; 4 Clinical Laboratory and Molecular Diagnostic, IRCCS INRCA, Ancona, Italy

**Keywords:** Lung ultrasound, Heart failure, NT-proBNP, Older adults

## Abstract

**Aim:**

Diagnosing and classifying heart failure (HF) in the oldest-old patients has technical and interpretation issues, especially in the acute setting. We assessed the usefulness of both N-terminal pro-brain natriuretic peptide (NT-proBNP) and lung ultrasound (LUS) for confirming HF diagnosis and predicting, among hospitalized HF patients, those with reduced ejection fraction (HFrEF).

**Methods:**

We performed a cross-sectional study on 148 consecutive patients aged ≥ 80 years admitted to our Internal Medicine and Geriatrics ward with at least one symptom/sign compatible with HF and NT-proBNP ≥ 125 pg/mL. We measured serum NT-proBNP levels and performed LUS and transthoracic echocardiography (TTE) on admission before diuretic therapy. We divided our cohort into three subgroups according to the left ventricular ejection fraction (LVEF): reduced (LVEF ≤ 40%), mildly-reduced (LVEF = 41-49%) and preserved (LVEF ≥ 50%).

**Results:**

The mean age was 88±5 years. Male prevalence was 42%. Patients with HFrEF were 19%. Clinical features and laboratory parameters did not differ between the three subgroups, except for higher NT-proBNP in HFrEF patients, which also had a higher number of total B-lines and intercostal spaces of pleural effusion at LUS. Overall, NT-proBNP showed an inverse correlation with LVEF (r = -0.22, *p* = 0.007) and a direct correlation with age, total pulmonary B-lines, and intercostal spaces of pleural effusion. According to the ROCs, NT-proBNP levels, pulmonary B-lines and pleural effusion extension were poorly predictive for HFrEF. The best-performing cut-offs were 9531 pg/mL for NT-proBNP (SP 0.70, SE 0.50), 13 for total B-lines (SP 0.69, SE 0.85) and one intercostal space for pleural effusion (SP 0.55, SE 0.89). Patients with admission NT-proBNP ≥ 9531 pg/mL had a 2-fold higher risk for HFrEF (OR 2.5, 95% CI 1.3-4.9), while we did not find any association for total B-lines ≥ 13 or pleural effusion ≥ 1 intercostal space with HFrEF. A significant association with HFrEF emerged for the combination of NT-proBNP ≥ 9531 pg/mL, total B-lines ≥ 13 and intercostal spaces of pleural effusion ≥ 1 (adjusted OR 4.3, 95% CI 1.5-12.9).

**Conclusions:**

Although NT-proBNP and LUS help diagnose HF, their accuracy in discriminating HFrEF from non-HFrEF was poor in our real-life clinical study on oldest-old hospitalized patients, making the use of TTE still necessary to distinguish HF phenotypes in this peculiar setting. These data require confirmation in more extensive and longer prospective studies.

**Supplementary Information:**

The online version contains supplementary material available at 10.1186/s12877-024-04977-4.

## Introduction

Heart failure (HF) is a leading cause of hospitalization and death among older adults worldwide [1]. In this population, classical clinical presentation features and standard radiologic imaging, well captured by the major and minor Framingham HF criteria [2], often lack accuracy, limiting early HF diagnosis and adequate treatment [3]. Cognitive impairment, prolonged bed rest, low collaboration, and comorbidities are significant confounders in this setting. Considering these issues, serum biomarkers of cardiac overload, such as N-terminal pro-brain natriuretic peptide (NT-proBNP), are helpful because of their high predictive diagnostic and prognostic value in HF patients of all ages [4–6]. On the other side, lung ultrasound (LUS) has emerged as an effective and simple tool for distinguishing between causes of acute and chronic dyspnea [7]. Unfortunately, objectifying and classifying a cardiac dysfunction, especially in the acute setting, remains a prerogative of trans-thoracic echocardiography (TTE), which is not always available or accessible to perform, especially in older patients. TTE provides information on the pericardium, cardiac chambers volumes and geometry, valve morphology and function and ventricular performance, allowing the current clinical classification of reduced, mildly-reduced and preserved ejection fraction (HFrEF, HFmrEF and HFpEF, respectively) [8]. Recognizing HFrEF is pivotal because its therapeutic approach is broader and based on more significant scientific evidence [9]. Specific devices and procedures, such as intracardiac defibrillator (ICD) or cardiac resynchronization therapy (CRT), and innovative drugs, such as sacubitril/valsartan, are exclusively or predominantly indicated in the management of HFrEF [10].

In this real-life clinical study conducted in an acute setting, we assessed the usefulness of NT-proBNP and LUS in identifying oldest-old patients with HF, focusing on their ability to predict those at higher risk of HFrEF.

## Materials and methods

### Study population and protocol

We conducted a cross-sectional study on patients consecutively admitted to the Internal Medicine and Geriatrics Unit (IRCCS INRCA, Ancona, Italy) from January 2022 to March 2023 for acute medical conditions and concomitant suspected heart failure (HF). We took into account the following inclusion criteria: i) patients aged ≥ 80 years; ii) referral to the Emergency Department (ED) with at least a symptom or sign compatible with HF based on the Framingham HF criteria (dyspnoea, ankle oedema, jugular vein distension, pulmonary rales, pleural effusion, cardiomegaly, pulmonary oedema); iii) admission NT-proBNP levels ≥ 125 pg/mL, as recommended by the 2021 ESC Guidelines for the Diagnosis and Treatment of Acute and Chronic Heart Failure [8]. We excluded patients with acute coronary syndrome, end-stage renal disease (ESRD) or dialysis, decompensated cirrhosis, active cancer, established interstitial lung disease (interstitial pneumonia, fibrosis), and patients having conditions with a life expectancy of less than one year. All participants gave their informed consent, and clinical investigations were conducted according to the principles expressed in the Declaration of Helsinki and its later amendments. This study was approved by the local institutional ethics committee (Comitato Etico INRCA). We collected clinical history, anthropometrics and laboratory parameters, basic activities of daily living (BADL) score, and pharmacological treatments on admission. Furthermore, we dosed admission NT-proBNP levels before starting or increasing diuretic therapy or modifying/introducing other HF therapies. We performed a complete LUS and TTE at admission, according to the best clinical practice. Both examinations were performed by well-trained and certified operators (CDP, PG). For the analyses, we divided our population into three subgroups according to the left ventricular ejection fraction (LVEF) measured during TTE: HFrEF (LVEF ≤ 40%), HFmrEF (LVEF = 41-49%) and HFpEF (LVEF ≥ 50%) [8]. Given the aim of our study, which focused on the prediction of HFrEF, the distribution of the study population and the significant impact on the clinical approach of HFrEF [8], we also decided to analyse the study population after dividing it into two subgroups: HFrEF (LVEF ≤ 40%) and non-HFrEF (LVEF > 40%).

### NT-proBNP assay

After blood sampling, NT-proBNP was measured using Elecsys proBNPII electrochemiluminescence immunoassay in a Cobas e601 immunoassay Roche analyzer. This assay contains two monoclonal antibodies that recognize epitopes located in the N-terminal part (1–76) of proBNP (1–108) [11].

### Transthoracic echocardiography

According to the American Society of Echocardiography [12], a trained physician performed the TTE at rest using a Vivid-7 (General Electric, Norway) ultrasound machine. Left ventricular (LV) volume, geometry, and mass (LVM) were assessed from the parasternal long-axis projection and adjusted for the body surface area (BSA). The modified biplane Simpson's method calculated the LVEF. Diastolic function was determined from the pattern of mitral inflow (early E wave and atrial a wave) acquired by pulsed Doppler and the average E/e' ratio after measuring septal and lateral mitral annular velocity (septal and lateral e') by tissue Doppler imaging (TDI). The right ventricle (RV) structure and function assessment was performed through the measure of tricuspid annular plane systolic excursion (TAPSE) and TD-derived tricuspid lateral annular systolic velocity (S'). Estimated systolic pulmonary artery pressure (PAPs) was derived from the tricuspid regurgitant jet together with an estimate of the right atrial pressure (RAP) based on inferior vena cava (IVC) size and breathing-related collapse.

### Lung ultrasound

LUS indirectly evaluated pulmonary interstitial involvement by detecting pulmonary B-lines (vertical hyperechoic laser-like artefacts arising from the pleura). Eight chest sites were scanned bilaterally, and the total bilateral B-lines were recorded. The interstitial syndrome was defined as three or more B-lines per intercostal space in at least two fields per side [13]. Also, basolateral lung scanning was performed to assess the presence of pleural effusion. If present, we measured the extension of the pleural effusion in terms of the cranial-caudal total number of intercostal spaces involved.

### Statistical analysis

Continuous variables were checked for normality and expressed as mean ± standard deviation or median and interquartile range (IQR) for markedly skewed variables. Categorical variables were expressed as numbers and percentages. Correlations between non-parametric variables were assessed by Spearman's 2-tailed method. Comparisons between variables were performed using the Student's t-test, the Mann-Whitney U-test, the analysis of variance (ANOVA) and the Kruskal-Wallis test. The chi-squared test was used for the comparison between categorical variables. The diagnostic utility of NT-proBNP levels and LUS parameters in detecting HFrEF was determined using receiver-operating characteristic (ROC) curves. The best threshold was obtained by selecting the ROC curve point that maximized sensitivity and specificity (Youden index). Logistic regression and ordinal regression were used to test the independent associations between covariates and the outcome. A *p*-value < 0.05 was considered statistically significant. All statistical analyses were conducted with SPSS version 23 [SPSS Inc., Chicago, IL, USA], Microsoft Windows version.

## Results

A total of 148 patients were enrolled in our study; 62 patients (42%) were males, mean age was 88 ± 5 years, and mean BMI was 25 ± 4 Kg/m^2^. The most prevalent comorbidities were hypertension (90%), chronic kidney disease (CKD) (53%), chronic HF (49%), and atrial fibrillation (AF) (44%). Framingham HF criteria were positive in 75% of patients. The average BADL score was 3 ± 2 points. The overall median NT-proBNP level was 7051 pg/mL (IQR 2541-15249). LUS detected pulmonary interstitial syndrome in 31% of patients, with an overall median of 9 total B-lines (IQR 6 - 15). Pleural effusion was detected in 68% of LUS examinations, with a median extension of 3 intercostal spaces (IQR 1-4). According to the TTE findings, 28 patients (19%) had HFrEF, 27 patients (18%) had HFmrEF, and 93 patients (63%) had HFpEF. No significant differences emerged between HFmrEF and HFpEF regarding the main clinical, laboratory and ultrasound parameters, especially NT-proBNP levels, total pulmonary B-lines and intercostal spaces of pleural effusion. On the other hand, the HFrEF subgroup had higher NT-proBNP levels, total B-lines and intercostal spaces of pleural effusion at LUS, and higher left ventricular mass index (LVMi). Clinical, laboratory parameters and ultrasound measurements, according to LVEF-based categories, are reported in Table 1.

**Table 1:** Main clinical characteristics, laboratory and ultrasound parameters according to the three LVEF-based categories

**Clinical parameters**	**Total** **(*n* = 148)**	**HFrEF** **(*n* = 28)**	**HFmrEF** **(*n* = 27)**	**HFpEF** **(*n*=93)**	***p* for comparison between subgroups**
**Age (years)**	88 ± 5	88 ± 5	88 ± 5	88 ± 5	0.934
**Sex (males, %)**	62 (42)	14 (50)	12 (44)	36 (38)	0.545
**BMI (Kg/m**^**2**^**)**	25 ± 4	25 ± 4	25 ± 4	25 ± 5	0.985
**Hypertension (%)**	134 (90)	27 (96)	23 (85)	84 (90)	0.360
**Atrial fibrillation (%)**	64 (44)	11 (40)	14 (52)	40 (43)	0.617
**Diabetes Mellitus (%)**	31 (21)	5 (18)	6 (22)	20 (21)	0.902
**Ischemic Heart Disease (%)**	49 (33)	12 (43)	6 (22)	31 (33)	0.266
**Chronic Heart Failure (%)**	72 (48)	16 (57)	11 (40)	45 (48)	0.475
**COPD (%)**	47 (32)	7 (25)	7 (26)	33 (35)	0.447
**Anemia (Hb < 12 g/dL) (%)**	65 (44)	9 (32)	11 (40)	45 (48)	0.295
**Non-end stage CKD (%)**	78 (53)	18 (64)	13 (48)	47 (50)	0.385
**Cognitive impairment (%)**	61 (42)	10 (36)	14 (52)	37 (40)	0.430
**Loop diuretic (%)**	103 (70)	21 (75)	20 (74)	62 (66)	0.601
**Beta-blocker (%)**	86 (58)	17 (60)	15 (55)	54 (58)	0.907
**RAASi (%)**	77 (52)	15 (53)	11 (40)	51 (55)	0.340
**Framingham HF + (%)**^**a**^ **Laboratory parameters**	112 (76)	24 (86)	21 (78)	67 (72)	0.322
**NT-proBNP (pg/mL)**	7051 (2541-15249)	9983 (5314-15597)	5913 (2980-15556)	5643 (2099-10400)	**0.045**
**eGFR (ml/min)**	46 ± 22	40 ± 20	49 ± 23	47 ± 22	0.133
**LUS parameters**					
** B-lines (n)**	12 (6-18)	16 (13-19)	12 (6-18)	12 (6-15)	**< 0.001**
** IS of pleural effusion (n)**	3 (1-4)	3 (2-4)	1 (0-3)	1 (0-3)	**0.016**
**TTE parameters**					
** LVEF (%)**	50 ± 11	30 ± 7	45 ± 1	57 ± 5.4	**< 0.001**
** RWT**	0.49 ± 0.14	0.48 ± 0.16	0.51 ± 0.16	0.48 ± 0.12	0.685
** LVMi (g/m**^**2**^**)**	106 ± 29	120 ± 40	110 ± 30	102 ± 25	**0.027**
** LAVi (ml/m**^**2**^**)**	44 ± 19	50 ± 27	40 ± 14	43 ± 16	0.126
** E/E’**	13 ± 4	12 ± 5	14 ± 5	13 ± 4	0.336
** TRV (m/s)**	2.8 ± 0.5	2.6 ± 0.5	2.9 ± 0.3	2.8 ± 0.5	0.462
** TAPSE (mm)**	20 ± 5	17 ± 4	19 ± 4	20 ± 5	0.059
** IVC (mm)**	20 ± 4	21 ± 5	20 ± 5	19 ± 4	0.401
** PAPs (mmHg)**	40 ± 12	37 ± 11	41 ± 14	41 ± 12	0.508
** Non-collapsible IVC (%)**	61 (41)	14 (50)	13 (48)	34 (37)	0.344

*HFrEF* Heart failure with reduced ejection fraction, *HFmrEF* Heart failure with mildly reduced fraction, *HFpEF* Heart failure with preserved ejection fraction, *BMI* Body mass index, *COPD* Chronic obstructive pulmonary disease, *Hb* Haemoglobin, *BADL* Basic activities of daily living, *CKD* Chronic kidney disease, *RAASi* Renin-angiotensin-aldosterone system inhibitors, *NT-proBNP* amino-terminal pro-brain-natriuretic peptide, *eGFR* estimated glomerular filtration rate, *LUS* Lung ultrasound, *IS* Intercostal spaces, *LVEF* Left ventricular ejection fraction, *RWT* Relative wall thickness, *LVMi* Left ventricular mass index, *LAVi* Left atrial volume index, *TRV* Tricuspid regurgitation velocity, *TAPSE* Tricuspid annular plane systolic excursion, *IVC* Inferior vena cava, *PAPs* systolic pulmonary arterial pressure

^a^Positivity is defined by two major or one major and two minor criteria. Major criteria: Paroxysmal nocturnal dyspnoea; Neck veins distention; Rales; Radiographic cardiomegaly (increasing heart size on chest radiography); Acute pulmonary oedema; S3 gallop; Increased central venous pressure (>16 cm H2O at right atrium); Hepatojugular reflux; Weight loss >4.5 kg in 5 days in response to treatment. Minor criteria: Bilateral ankle oedema; Nocturnal cough; Dyspnoea on ordinary exertion; Hepatomegaly; Pleural effusion; Decrease in vital capacity by one-third from maximum recorded; Tachycardia (heart rate>120 beats/min)

### Correlations between NT-proBNP, LUS and TTE parameters

NT-proBNP levels showed a direct correlation with age (r = 0.25, *p* = 0.002), total number of pulmonary B-lines (r = 0.18, *p* = 0.025), pleural effusion extension (r = 0.26, *p* = 0.001), and positivity of the Framingham HF criteria (r = 0.14, *p* = 0.026). An inverse correlation was found between NT-proBNP, LVEF (r = - 0.22, *p* = 0.007) and eGFR (r = - 0.33, *p* < 0.001). The total number of B-lines and the extension of pleural effusion were inversely correlated with LVEF (r = - 0.25, *p* = 0.002 and r = - 0.22, *p* = 0.008, respectively) and directly related with age and positivity of the Framingham HF criteria (r = 0.19, *p* = 0.027 and r = 0.21, *p* = 0.011, respectively).

### ROCs

**Fig. 1:**
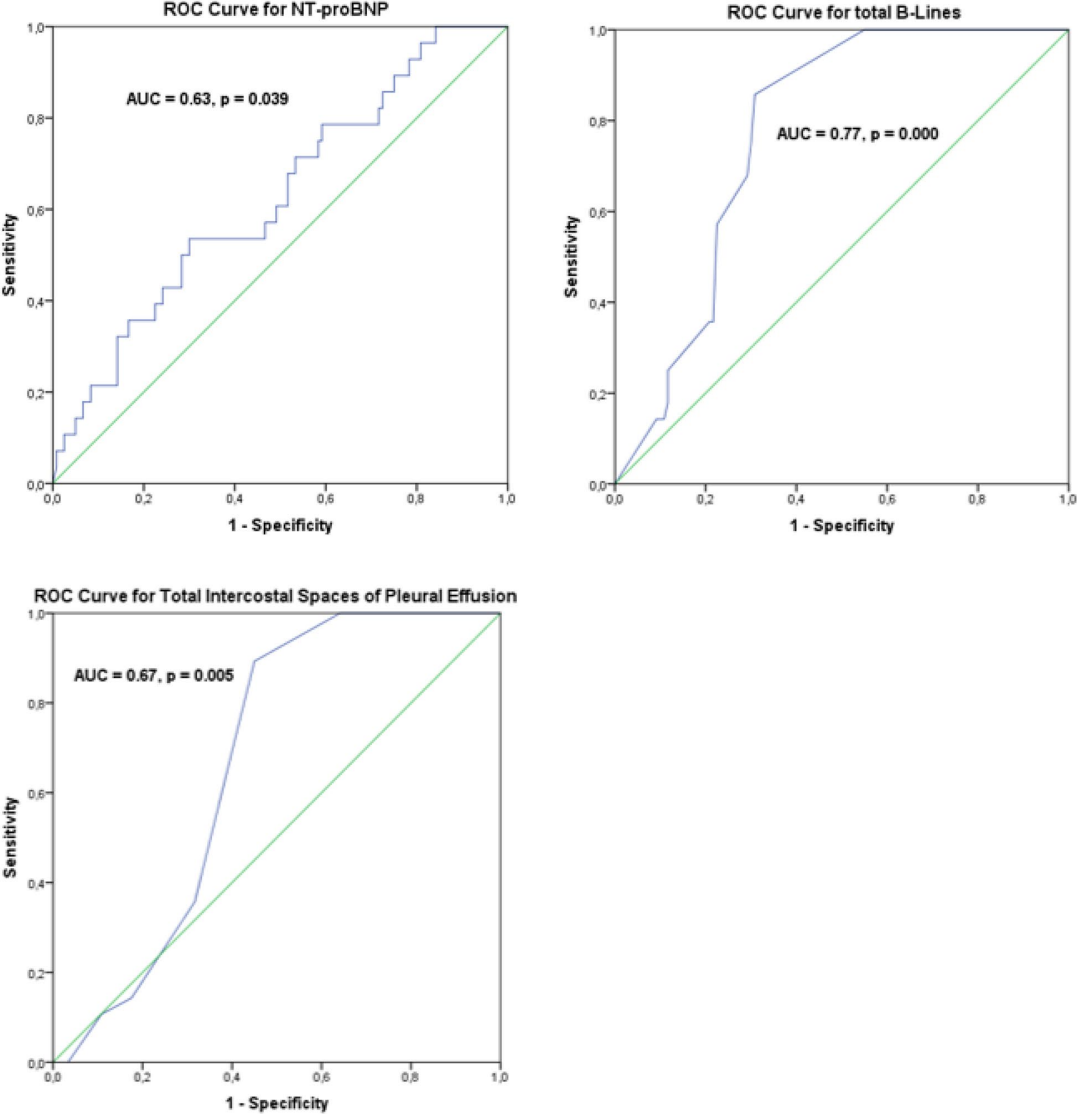
ROCs for NT-proBNP, total B-lines and intercostal spaces of pleural effusion. The figure shows the accuracy of NT-proBNP serum concentrations, number of pulmonary B-lines and spaces of pleural effusion in identifying patients with HFrEF (vs non-HFrEF). Each parameter was significantly able to identify HFrEF but with low-moderate accuracy, as reported by the AUC of the ROC curves

According to the ROCs, NT-proBNP levels (AUC 0.63, *p* = 0.039), pulmonary B-lines (AUC 0.77, *p* = 0.001) and pleural effusion extension (AUC 0.67, *p* = 0.005) can predict HFrEF (vs non-HFrEF). The optimal cut-offs for HFrEF prediction were the following: 9531 pg/mL for NT-proBNP (SP 0.70, SE 0.50), 13 for the number of total B-lines (SP 0.69, SE 0.85) and 1 for the number of intercostal spaces of pleural effusion (SP 0.55, SE 0.89) (Fig. 1).

### Associations between NT-proBNP, B-lines, and extension of pleural effusion with HFrEF diagnosis

The optimal cut-offs found in the ROCs were used to investigate the association with HFrEF diagnosis.

Association with HFrEF diagnosis was found for an NT-proBNP value ≥ 9531 pg/mL but not for B-lines ≥ 13 and intercostal spaces of pleural effusion ≥ 1. After considering the simultaneous presence of NT-proBNP and LUS variables above the cut-offs, a significant increase in HFrEF risk emerged. However, it remained absent in most patients identified as HFrEF (25% in the HFrEF group vs 6% in non-HFrEF). The multivariable analysis demonstrated that the association of the combination of NT-proBNP as a dichotomous variable and LUS parameters with HFrEF diagnosis remained valid even after adjustment for age, sex and eGFR. Table 2 shows the risk of being included in a lower LVEF subgroup, down to the HFrEF.

**Table 2:** Odds-ratios for being included in a lower LVEF subgroup, down to the HFrEF

	**HFrEF (%)**	**HFmrEF (%)**	**HFpEF (%)**	**OR (95% CI)**	**OR**^**a**^** (95% CI)**
**Total**	*n* = 28 (19)	*n* = 27 (18)	*n* = 93 (63)	-	-
**NT-proBNP < 9531 pg/mL**	13 (46)	16 (59)	68 (73)	Ref.	Ref.
**NT-proBNP ≥ 9531 pg/mL**	15 (54)	11 (41)	25 (27)	**2.5 (1.3-4.9)**	-
**B-lines < 13**	16 (57)	16 (59)	67 (72)	Ref.	Ref.
**B-lines ≥ 13**	12 (43)	11 (41)	26 (28)	1.8 (0.9-3.5)	-
**Intercostal spaces < 1**	7 (25)	10 (37)	33 (36)	Ref.	Ref.
**Intercostal spaces ≥ 1**	21 (75)	17 (63)	60 (64)	1.3 (0.7-2.6)	-
**Combination -**	21 (75)	25 (93)	88 (95)	Ref.	Ref.
**Combination +**	7 (25)	2 (7)	5 (5)	**4.4 (1.5-12.9)**	**4.3 (1.5-12.9)**

OR^a^ adjusted for age, sex and eGFR

*HFrEF* Heart failure with reduced ejection fraction, *HFmrEF* Heart failure with mildly reduced ejection fraction, *HFpEF* Heart failure with preserved ejection fraction, *NT-proBNP* amino-terminal pro-brain natriuretic peptide

## Discussion

Diagnosing HF in very old patients with comorbidities represents a daily challenge for clinicians in both the ED and medical wards. Considering dyspnoea as the most frequent symptom of HF presentation, comorbidities such as COPD, AF, CKD, and anemia may complicate the diagnosis, thus severely limiting early and accurate HF recognition and adequate treatment. Distinguishing HFrEF from non-HFrEF, characterized by different pathophysiologic aspects and treatments, superimposes a challenge within a challenge [14]. Despite being the diagnostic gold standard, TTE is not widely available and needs adequate clinician training, time, and compliance of the patients, which may not be easily obtained in the older population. In our analysis, 42% of patients suffered from a cognitive decline, and the average BADL scale scored 3, indicating a moderate-to-severe reduction in personal autonomy. These conditions and comorbidities could compromise the feasibility and accuracy of the TTE. If it is not easy to refer these older patients for TTE from a primary care setting, there can be several challenges in performing a TTE, even in the hospital setting. Indeed, the majority of oldest old hospitalized HF patients with frailty and comorbidities are admitted to internal medicine/geriatric wards, where TTE availability can be low, rather than specialized cardiology wards, where TTE is usually available [15]. Conversely, LUS is widely available and used in internal medicine/geriatric wards [16, 17]. A role could also be played by what has been defined as *ageism*, an increasingly recognized form of bias involving stereotypes, prejudice, and discrimination directed toward people based on their age [18]. Indeed, age was a negative predictor for echocardiographic evaluation among older patients in previous studies [19]. A retrospective survey of 116 patients with a median age of 86 years and an established diagnosis of HF found that those who did not undergo TTE during the hospitalization were older and frailer compared to those who received the echocardiographic evaluation [3]. According to mere technical considerations, LUS is usually more rapid and easy to perform than TTE, especially in the acute setting where non-cardiologists or non-adequately trained physicians manage older patients with suspected HF [20, 21]. In addition to better operator training, TTE requires greater collaboration during the examination, which may not always be adequate in frail patients or those with cognitive decline. Therefore, the TTE image quality and interpretation are often more challenging in older adults because of the scarce collaboration, comorbidities and age-related changes [22].

Over the last decade, cardiac biomarkers have dramatically changed the management of HF patients. Natriuretic peptides (NPs), particularly B-type (BNP and NT-proBNP), have emerged as robust markers for diagnosis, prognosis and management of HF patients [23]. Although most studies on plasma NT-proBNP concentrations only included individuals with a mean age < 70 years, most HF patients are older in the daily clinical practice. Several studies have demonstrated that age and cardio-renal and pulmonary comorbidities may increase NT-proBNP levels, lowering the accuracy of HF diagnosis [24]. Thus, in the setting of HF diagnosis in patients aged >75 years, a diagnostic cut-off of 1800 pg/mL has been proposed [25]. NP levels have maintained their negative predictive value, which helps rule out cardiogenic dyspnoea when values are below specific cut-offs [26, 27]. In this study, we chose to enrol older patients with NT-proBNP ≥ 125 pg/ml according to what has been recommended by the 2021 ESC Guidelines [8] for guiding HF diagnosis and the decision to perform an echocardiographic evaluation to objectify the presence of heart disease. In our acute setting of older patients with a mean age of 88 years, the NT-proBNP levels are usually much higher [6]. Accordingly, the median NT-proBNP level in our older population was 7051 pg/mL (IQR 2541-15249), about four times higher than 1800 pg/ml. NT-proBNP is the most reliable biomarker for the diagnosis, prognosis and management of HF, thanks to its biological stability and non-interference with neprilysin, currently the target of sacubitril/valsartan [28, 29]. In our study, no enrolled patients were taking this drug. Previous studies found that NT-proBNP correlated directly with LV end-diastolic dimension (LVEDD) and volumes and inversely with LVEF, along with significant differences between HFrEF and non-HFrEF patients [30]. In one report including more than 2000 patients with a median age of 73 years, the median NT-proBNP in the HFrEF group was 4580 pg/mL (IQR 2065–9765 pg/mL) vs 2900 pg/mL (IQR 2065–9765 pg/mL) in the non-HFrEF group (p < 0.01) [31]. Other studies confirmed this inverse association [32]. Likewise, a linear and inverse correlation emerged in our analysis between NT-proBNP levels and LVEF (r = - 0.22, p = 0.007), confirming that even older patients with HFrEF have higher NT-proBNP levels, reflecting significant myocardial stress and adverse remodelling [30]. Accordingly, our best NT-proBNP cut-off value for HFrEF was slightly above 9500 pg/mL. However, the ability of NT-proBNP to predict HFrEF was weak (AUC 0.63, p < 0.05), and the threshold lacked accuracy (70% specificity, 50% sensibility) to recognize HFrEF. The NT-proBNP cutpoint of 9531 pg/mL found in our study derives from the Youden index of the ROC. However, its low sensitivity and specificity do not allow its use in clinical practice per se, and our small sample of HFrEF patients could have driven this high cut-off. This value is close enough to the median value in this oldest population with high NT-proBNP values overall. Currently, the aged-adjusted NT-proBNP cut-off considered as a discriminant for acute HF in subjects over 75 years is 1800 pg/ml [33], and no further cut-offs have been proposed to date in even older subjects, probably due to a lack of evidence. Despite the limitation of the small sample size, we provided evidence from this poorly represented population, and our results suggest that even in the oldest-old population, NT-proBNP significantly and inversely correlates with LVEF. If our preliminary data will be confirmed by future studies on larger oldest-old samples, different NT-proBNP cutoffs may be needed in this peculiar population. Similarly, LUS seems to be as simple as an accurate method for assessing pulmonary congestion related to AHF in clinical practice. However, previous studies have documented the presence of B-lines in normal lungs among healthy geriatric patients, mainly isolated and confined to the lateral-basal areas [34]. Multiple bilateral B-lines and the detection of pleural effusion can increase the probability of a cardiogenic aetiology of dyspnoea [35]. Furthermore, the total number of B-lines has been associated with depressed LVEF [median B lines total number of 32 (IQR 27–38) in HFrEF group vs 30 (IQR 25–36) in non-HFrEF group; p = 0.05] in a prospective cohort of 250 patients with a median age of 80 years admitted with a diagnosis of HF [36]. Therefore, data concerning the usefulness of LUS for detecting HF and HFrEF in this particular population are scarce. As well as for NT-proBNP, we found pulmonary B-pattern and pleural effusion poorly predictive for HFrEF, with cut-offs of 13 total B-lines and 1 intercostal space, respectively, being far from a clinically acceptable diagnostic accuracy. Despite the low diagnostic performance, patients with NT-proBNP ≥ 9531 pg/mL had an almost three-fold higher risk of HFrEF. Moreover, after taking into account the subgroup characterized by NT-proBNP ≥ 9531 pg/mL, B-lines ≥ 13, and intercostal spaces of pleural effusion ≥ 1, the multivariable analysis reported a further significant higher risk of HFrEF, almost up to five times, even after adjustment for age, sex and renal function. However, it is essential to note that this triple combination was absent in most of the identified HFrEF patients in our study (Table 2). NT-proBNP and LUS signs reflect cardiovascular overload and pulmonary congestion, depending on volume status, diuretic therapy, and renal function. In our study, no difference was found in the prevalence of loop diuretic therapy and mean eGFR between groups. The low diagnostic accuracy of NT-proBNP and LUS signs found in our investigation could reflect the complexity behind the development of congestion in AHF, mainly driven by a fluid redistribution and a fluid accumulation, that often coexist in both HFrEF and non-HFrEF, leading to comparable haemodynamic congestion and overload [37, 38].

### Study limits

Our study has several limitations that need to be pointed out. First, the small sample size, especially regarding the subgroup of HFrEF patients (n° 28), may have led to statistical biases affecting the adequate evaluation of the diagnostic performance of the combined variables. Non-HFrEF is more prevalent than HFrEF in the older population due to different aetiologies and ageing-related mechanisms [39], especially in a population with a mean age of 88 years. Therefore, further studies on larger populations are needed. A post-hoc power analysis based on the ROCs for the total B-lines and the total intercostal spaces of pleural effusion demonstrated that the canonical 80% power was reached for both variables. Despite such limitations, this observational study represents a precious opportunity to investigate the oldest-old populations that are otherwise usually excluded, paving the way for future studies. Moreover, we classified HF based on LVEF evaluated on TTE because it is the most used method in daily clinical practice, having pivotal implications, especially regarding prognosis and drug therapy. Therefore, assessing the role of NT-proBNP and LUS in different subtypes of HF identified using classification criteria other than LVEF (i.e. based on aetiology, such as hypertensive vs ischemic vs valvular) was out of the purpose of our study and specifically designed investigation should be carried out to address this issue. We cannot provide data about inter and intra-observer variability coefficients between operators of ultrasound evaluations. The lack of these quality parameters is common in previous “real-life” studies, especially in acute clinical settings [39]. Of course, we could not exclude biases of ultrasound measures related to variability [40]. However, regarding the precision of LUS in detecting interstitial involvement (B-lines), which we mainly assessed in our study, previous data have been consistent with a high grade of interobserver agreement [41].

## Conclusion

Identifying simple and valuable tools that may help physicians in the ED or internal medicine/geriatric wards to intercept those older patients at higher risk of HFrEF, thus deserving further investigations and/or specific treatments, could be very useful in clinical practice. Accurate diagnosis and classification of HF patients according to echocardiographic evaluation still preserve a role in guiding the clinician to optimize the pharmacological and non-pharmacological therapy to reduce symptoms recurrence, hospitalization and mortality effectively. In our real-life clinical study, we tested the use of NT-proBNP and LUS for identifying HFrEF among oldest-old hospitalized HF patients, showing that, although an association is present, their accuracy is low in this population. This low diagnostic accuracy does not allow us to avoid the systematic use of TTE to distinguish HF phenotypes in this peculiar setting. On the other side, the usefulness of these tools for HF diagnosis is evident in the literature. Our study is the first that investigated this issue. Therefore, our findings are not definitive and require further confirmation in more extended studies on larger populations.

### Supplementary Information

**Additional file 1.** **Supplemental Table S1.** Main clinical characteristics, laboratory and ultrasound parameters according to HFrEF or non-HFrEF. **Supplemental Table S2.** Odds-ratios for HFrEF diagnosis.

## Data Availability

The datasets used and analysed during the current study are available from the corresponding author upon reasonable request.
