# Supplementary material for: Role of NT-proBNP and lung ultrasound in diagnosing and classifying heart failure in a hospitalized oldest-old population: a cross-sectional study
Source: BMC Geriatr. 2024 Apr 20;24:354. doi: 10.1186/s12877-024-04977-4 (PMC11032608; doi:10.1186/s12877-024-04977-4)
Supplement: Supplementary file 1 — Additional file 1. Supplemental Table S1. Main clinical characteristics, laboratory and ultrasound parameters according to HFrEF or non-HFrEF. Supplemental Table S2. Odds-ratios for HFrEF diagnosis. [file 12877_2024_4977_MOESM1_ESM.pdf]

<u>Supplemental Table S1. Main clinical characteristics, laboratory and ultrasound parameters according to HFrEF or non-HFrEF</u>

**<u>Clinical parameters</u>**	**HFrEF (n = 28)**	**non-HFrEF (n = 120)**	**p**
**Age (years)**	88 ± 5	88 ± 5	0.927
**Sex (males, %)**	14 (50)	48 (40)	0.334
**BMI (Kg/m^2)**	25 ± 4	25 ± 5	0.879
**Hypertension (%)**	27 (96)	107 (90)	0.237
**Atrial fibrillation (%)**	11 (40)	54 (45)	0.583
**Diabetes Mellitus (%)**	5 (18)	26 (21)	0.656
**Ischemic Heart Disease (%)**	12 (43)	37 (31)	0.223
**Chronic Heart Failure (%)**	16 (57)	56 (47)	0.318
**COPD (%)**	7 (25)	40 (33)	0.394
**Anemia (Hb < 12 g/dL) (%)**	9 (32)	56 (46)	0.163
**Non-end stage CKD (%)**	18 (64)	60 (50)	0.173
**Cognitive impairment (%)**	10 (36)	51 (42)	0.511
**Loop diuretic (%)**	21 (75)	82 (68)	0.490
**Beta-blocker (%)**	17 (60)	69 (57)	0.855
**RAASi (%)**	15 (53)	62 (51)	0.949
**Framingham HF + (%)***	24 (86)	88 (73)	0.169
**<u>Laboratory parameters</u>**			
**NT-proBNP (pg/mL)**	9983 (5314-15597)	5700 (2286-11083)	***0.039***
**eGFR (ml/min)**	40 ± 20	48 ± 22	***0.050***
**<u>LUS parameters</u>**			
**B-lines (n)**	16 (13-19)	12 (6-15)	***0.001***
**IS of pleural effusion (n)**	3 (2-4)	1 (0-3)	***0.004***
**<u>TTE parameters</u>**			
**LVEF (%)**	30 ± 7	55 ± 7	***0.001***
**RWT**	0.48 ± 0.16	0.50 ± 0.13	0.067
**LVMi (g/m^2)**	120 ± 40	104 ± 26	***0.014***
**LAVi (ml/m^2)**	50 ± 27	43 ± 16	0.057
**E/E'**	12 ± 5	14 ± 5	0.356
**TRV (m/s)**	2.6 ± 0.5	2.8 ± 0.5	0.326
**TAPSE (mm)**	17 ± 4	19 ± 5	***0.031***
**IVC (mm)**	21 ± 5	19 ± 4	0.189
**PAPs (mmHg)**	37 ± 11	40 ± 12	0.254
**Non-collapsible IVC (%)**	14 (50)	47 (40)	0.230

HFrEF = heart failure with reduced ejection fraction, BMI = body mass index, COPD = chronic obstructive pulmonary disease, Hb = haemoglobin, BADL = basic activity of daily living, CKD = chronic kidney disease, RAASi = renin-angiotensin-aldosterone-system inhibitors, NT-proBNP = amino-terminal pro-brain-natriuretic peptide, eGFR = estimated glomerular filtration rate, LUS = lung ultrasound, IS = intercostal spaces, LVEF = left ventricular ejection fraction, RWT = relative wall thickness, LVMi = left ventricular mass index, LAVi = left atrial volume index, TRV = tricuspid regurgitation velocity, TAPSE = tricuspid annular plane systolic excursion, IVC = inferior vena cava, PAPs = systolic pulmonary arterial pressure.
**Positivity is defined by two major or one major and two minor criteria. Major criteria: Paroxysmal nocturnal dyspnoea; Neck vein distention; Rales; Radiographic cardiomegaly (increasing heart size on chest radiography); Acute pulmonary oedema; S3 gallop; Increased central venous pressure (>16 cm H2O at right atrium); Hepatojugular reflux; Weight loss >4.5 kg in 5 days in response to treatment. Minor criteria: Bilateral ankle oedema; Nocturnal cough; Dyspnoea on ordinary exertion; Hepatomegaly; Pleural effusion; Decrease in vital capacity by one-third from maximum recorded; Tachycardia (heart rate>120 beats/min).*

Supplemental Table S1 reports the same characteristics of the study population according to HFrEF or non-HFrEF patients.

Supplemental Table S2. Odds-ratios for HFrEF diagnosis

	HFrEF + (%)	HFrEF – (%)	OR (95% CI)	OR[1] (95% CI)
**Total**	n = 28 (19)	n = 120 (81)	-	-
**NT-proBNP < 9531 pg/mL**	13 (46)	84 (70)	Ref.	Ref.
**NT-proBNP ≥ 9531 pg/mL**	15 (54)	36 (30)	**2.7 (1.2-6.2)**	-
**B-lines < 13**	16 (57)	83 (69)	Ref.	Ref.
**B-lines ≥ 13**	12 (43)	37 (31)	1.6 (0.7-4)	-
**Intercostal spaces < 1**	7 (25)	43 (36)	Ref.	Ref.
**Intercostal spaces ≥ 1**	21 (75)	77 (64)	1.7 (0.6-4.2)	-
**Combination -**	21 (75)	113 (94)	Ref.	Ref.
**Combination +**	7 (25)	7 (6)	**5.3 (1.7-17)**	**5.4 (1.6-16)**

OR[1] = adjusted for age, sex and eGFR
HFrEF = heart failure with reduced ejection fraction, NT-proBNP = amino-terminal pro-brain natriuretic peptide

Supplemental Table S2 shows the risk of HFrEF compared to non-HFrEF.
